# Huge Leiomyomas Arising from Bilateral Uterine Remnants in a Mayer–Rokitansky-Küster-Hauser Syndrome Patient with Coexisting Myotonic Dystrophy Type 1: A Case Report and Literature Review

**DOI:** 10.1155/2023/5182889

**Published:** 2023-08-28

**Authors:** Yukihiro Azuma, Koji Yamamoto, Mei Matsumoto, Hiroki Nagata, Ikumi Wada, Keisuke Miyamoto, Fuminori Taniguchi

**Affiliations:** Department of Obstetrics and Gynecology, Tottori University Faculty of Medicine, Yonago 683-8504, Japan

## Abstract

Mayer-Rokitansky-Küster-Hauser syndrome (MRKHS) is a rare congenital anomaly of the genital tract. Since the secretion of sex hormones from the ovaries is preserved, leiomyomas and adenomyomas, which are estrogen-dependent diseases, may develop from the uterine remnant. In contrast, patients with myotonic dystrophy type 1 (DM1), the most common dystrophy in adults, are considered to be at high risk for benign tumors of the female reproductive system, such as uterine leiomyomas and ovarian cysts. A rare case of huge leiomyomas arising from bilateral uterine remnants in a woman with MRKHS with coexisting DM1 is presented. Her chief complaint was abdominal distension. On pelvic magnetic resonance imaging (MRI), two solid pelvic masses showing low signal intensity on T2-weighted imaging were seen. Both the uterine corpus and cervix were unclear, but bilateral ovaries were observed normally on MRI. Two uterine leiomyoma-like masses connected by a band of fibrous tissue were found by laparotomy. As with the MRI findings, the uterine cervix and vagina could not be detected macroscopically. Normal bilateral adnexa and round ligaments were identified. All of her symptoms improved after hysterectomy.

## 1. Introduction

Mayer-Rokitansky-Küster-Hauser syndrome (MRKHS) is a rare congenital anomaly of the genital tract. Although the etiology of MRKHS remains unexplained, the incidence of MRKHS has been estimated as approximately 1 in 4000–5000 female live births [1]. This disease is characterized by aplasia or hypoplasia of the uterus and upper 2/3 of the vagina. The patients present with a normal female appearance (pubic hair and breast development are Tanner stage 5) and normal 46 chromosomes, XX female karyotype. Renal, skeletal, ear, or cardiac malformations are known as the major extragenital anomalies. In most cases, the diagnostic trigger is eugonadal primary amenorrhea in adolescence. Since the secretion of ovarian hormones from the ovaries is normally preserved, there is a potential for the development of estrogen-dependent diseases, such as uterine leiomyomas and adenomyomas, from uterine remnants. However, there are few reports of leiomyoma cases developing from uterine remnants of MRKHS.

In contrast, myotonic dystrophy type 1 (DM1) is a multisystem, autosomal dominant disorder known for its skeletal muscle manifestations. The incidence of DM1 ranges between 0.5 and 18.1 per 100,000 populations [2]. DM1 is the most common dystrophy in adults, and is caused by trinucleotide repeat expansion of cytosine–thymine–guanine (CTG) in the 3′-untranslated region (3′-UTR) of dystrophy protein kinase gene (DMPK) on chromosome 19q 13.3. It has been reported that patients with DM1 are at high risk for benign tumors of the female reproductive system, such as uterine leiomyomas [3]. Expanded CTG repeats in tumor tissue are considered to increase the risk for tumorigenesis through the abnormal splicing of mRNA transcription [4].

A rare case of huge leiomyomas arising from bilateral uterine remnants in an MRKHS patient with coexisting DM1 is presented.

## 2. Case Report

A 50-year-old woman visited our institution due to a complaint of abdominal distension. She had a history of primary amenorrhea and was previously diagnosed with DM1 based on clinical features, such as progressive muscle weakness of the limbs. On genetic testing, her CTG repeat length in the 3′-UTR of DMPK exceeded 1200 repeats.

**Figure 1:**
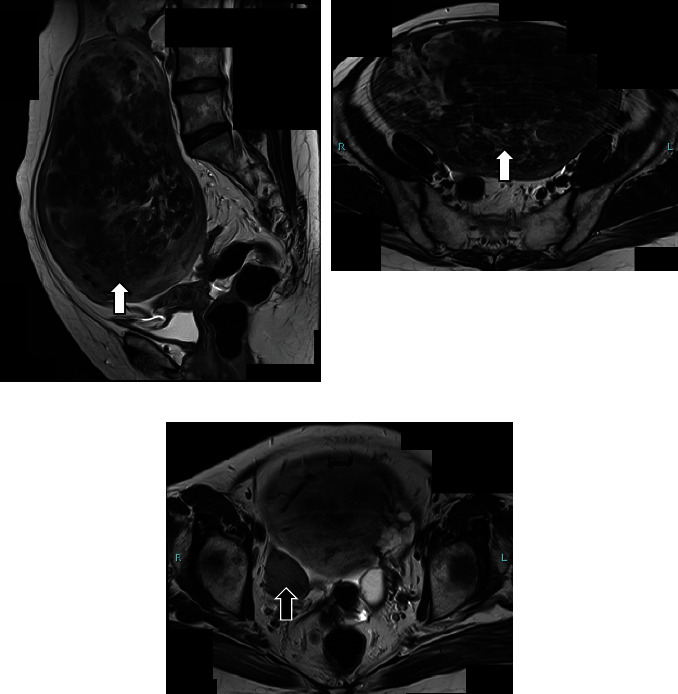
MRI findings on T2-weighted imaging. MRI shows two solid pelvic masses. (a) White and (b) black arrows indicating the different masses.

**Figure 2:**
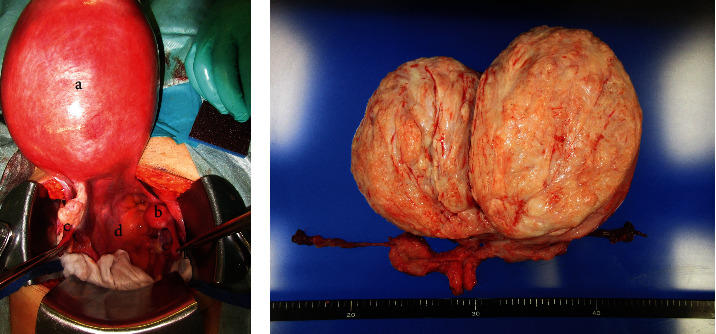
Huge leiomyomas arise from the uterine remnant of MRKHS. (a) Two masses, connected by a band of fibrous tissue, are observed. (A: a larger mass arising from the left uterine remnant; B: a small mass arising from the right uterine remnant; C: normal bilateral adnexa; and D: pouch of Douglas). (b) The specimen of the mass resembles uterine leiomyoma.

A gynecologic examination identified a blind-ending vagina and deficiency of the uterine cervix. Transrectal ultrasonography showed no uterine corpus and no cervix. On pelvic magnetic resonance imaging (MRI), two solid pelvic masses (19 and 4 cm in diameter, respectively), that showed low signal intensity on T2-weighted imaging were seen (Figure 1). The bigger mass grew beyond the sacral promontory. Although both the uterine corpus and cervix were unclear, normal bilateral ovaries were observed. No malformation of the urinary tract was found on drip infusion pyelography. The serum levels of estradiol and follicle-stimulating hormone were within normal ranges. All preoperative blood tests, including ovarian tumor markers and physical examinations, were normal. Based on the above results, she was diagnosed with MRKHS for the first time, and leiomyomas arising from bilateral uterine remnants were suspected preoperatively. Because the tumor was too bulky to treat with laparoscopic surgery, a total abdominal hysterectomy was performed. Two huge masses, like uterine leiomyomas, connected by a band of fibrous tissue were observed (Figure 2). The uterine cervix and vagina could not be observed clearly, whereas bilateral adnexa and round ligaments were identified. The total weight of the excised tissues was 1750 g. No complications occurred perioperatively, and all symptoms, including abdominal distension and frequent urination, were completely relieved after surgery. Histological examination of the masses growing from bilateral uterine remnants showed the findings of leiomyoma. In the tumors and fibrous band connecting the uterine remnants, there was no glandular epithelium.

## 3. Discussion

This is the first report of huge leiomyomas arising from bilateral uterine remnants in a woman with MRKHS with coexisting DM1. MRKHS is generally classified into two types according to the degree of morphological abnormality [5]. Type I MRKHS shows complete uterine aplasia in the presence of two rudimentary horns linked by a salpinx. Type II MRKHS is characterized by symmetric or asymmetric uterine hypoplasia, involving aplasia of one or two horns, or by a size difference between the two horn rudiments. This patient had two rudimentary uterine remnants, cervical agenesis, and vaginal hypoplasia, corresponding to type I MRKHS. The American Fertility Society Classification of 1988 has been the most common to classify Müllerian anomalies. However, this was insufficient for MRKHS patients because it lacked the assessment of vaginal and cervical anomalies. The American Society for Reproductive Medicine published a modified classification for more accurate diagnosis in 2021. According to this classification, MRKHS was classified as Müllerian agenesis [6].

Beecham et al. reported on MRKHS with a myoma first in 1977 [7]. Based on our literature review (22 MRKHS patients with remnant leiomyomas) [8–28], the median diameter of leiomyomas is 10 cm (range 4.5–19 cm), and the median age of the patients is 42 years (range 25–70 years; Table 1). The present case had one of the largest leiomyomas arising from uterine remnants of MRKHS of the previous reports. For precise imaging when genital tract anomalies are suspected, ultrasonography has limitations for accurate diagnosis of Müllerian duct anomalies, including MRKHS. In contrast, MRI has nearly 100% accuracy in the diagnosis of Müllerian duct anomalies [29] and identification of rudimentary uteri and ovaries in MRKHS patients [30], because T2-weighted imaging can depict pelvic soft tissues, such as the uterus and vagina.

Several case reports have suggested that DM1 patients are at high risk for benign and malignant tumors, as typified by pilomatrixoma. The data regarding DM1 patients (*n* = 409) enrolled in the UK Myotonic Dystrophy Patient Registry demonstrated that tumors of the female reproductive system were the most common benign tumors [3]. Malignant tumors derived from the female genital tract were not reported in that study. Moreover, a larger-size study demonstrated that the hazard ratio (HR) for uterine leiomyoma was elevated in DM1 females related to DM1-free individuals (HR = 2.7; 95% confidence interval = 1.22–5.88) [31]. Although the molecular mechanism is not determined, some findings were as follows: (i) CTG repeat in several tumors of DM1 patients [32]; and (ii) cells with larger CTG repeat expansions had a growth advantage over those with smaller expansions in cultured normal lymphoblastoid cell lines [33]. In addition, Khajavi et al. suggested that this expansion of the CTG repeat was attributable to increased cell proliferation *via* ERK1/2. In the review relevant to neoplasms in DM, Mueller et al. suggested that abnormal accumulation of *β*-catenin via the Wnt/*β*-catenin signaling pathway may play an important role in DM-related tumorigenesis [34]. In the presence of Wnt signaling, the phosphorylation and degradation of *β*-catenin are blocked, therefore, the accumulation of *β*-catenin in the nucleus is led. The accumulated *β*-catenin promotes transcriptional activation of c-Myc and cyclin D1, consequently leading to cell proliferation. On the other hand, it has been previously reported that CTNNB1 (the gene of *β*-catenin) is aberrantly expressed in uterine leiomyoma tissue compared with normal myometrium [35]. These findings indicate that *β*-catenin may play a causal role in uterine leiomyoma development in DM1 patients. Furthermore, research is needed to elucidate the etiology associated with tumor development in DM1. In the present case, the coexisting DM1 may have contributed to the enormous growth of the leiomyomas of the uterine remnants. When a DM1 patient complains of a lower abdominal mass, uterine myoma should be kept in mind.

## Figures and Tables

**Table 1:** Leiomyomas arising from uterine remnants in MRKHS patients.

No.	Authors	Year	Country	Age (y)	Diameter of leiomyoma (cm)
1	Rhee CS et al. [8]	1999	South Korea	49	10
2	Tsin DA et al. [9]	2000	Astoria	36	8.5
3	Edmonds DK et al. [10]	2003	England	70	10
4	Jadoul et al. [11]	2004	Belgium	42	10
5	Deligeoroglou E et al. [12]	2004	Greece	38	5.9
6	Deligeoroglou E et al. [12]	2004	Greece	42	4.8
7	Lamarca M et al. [13]	2009	Spain	35	5
8	Fukuda J et al. [14]	2010	Japan	50	7
9	Rawat KS et al. [15]	2013	India	35	16
10	Kundu K et al. [16]	2014	USA	40	9
11	Vidyashree PG et al. [17]	2015	India	40	10
12	Kulkarni MM et al. [18]	2015	India	25	5
13	Girma WM et al. [19]	2015	Ethiopia	40	18
14	Sharma R et al. [20]	2017	India	45	18
15	Amaratunga T et al. [21]	2017	Canada	66	4.5
16	Blontzos N et al. [22]	2019	Greece	44	10
17	Jokimaa V et al. [23]	2020	Finland	47	5.5
18	Albahlol IA et al. [24]	2020	Egypt	45	15
19	Ibidapo-Obe O et al. [25]	2021	USA	47	19
20	Qiu S et al. [26]	2021	China	31	10
21	Jain N et al. [27]	2022	India	28	10
22	Parra CM et al. [28]	2022	USA	44	12

## References

[1] Varner R. E., Younger J. B., Blackwell R. E. (1985). Müllerian dysgenesis. *The Journal of Reproductive Medicine*.

[2] Theadom A., Rodrigues M., Roxburgh R., Balalla S., Higgins C., Bhattacharjee R. (2014). Prevalence of muscular dystrophies: a systematic literature review. *Neuroepidemiology*.

[3] Alsaggaf R., Wang Y., Marini-Bettolo C. (2018). Benign and malignant tumors in the UK myotonic dystrophy patient registry. *Muscle and Nerve*.

[4] D’Ambrosio E. S., Gonzalez-Perez P. (2023). Cancer and myotonic dystrophy. *Journal of Clinical Medicine*.

[5] Morcel K., Camborieux L., Guerrier D., Programme de Recherches sur les Aplasies Müllériennes (PRAM) (2007). Mayer-Rokitansky-Küster-Hauser (MRKH) syndrome. *Orphanet Journal of Rare Diseases*.

[6] Ludwin A., Tudorache S., Martins W. P. (2022). ASRM Müllerian anomalies classification 2021: a critical review. *Ultrasound in Obstetrics and Gynecology*.

[7] Beecham C. T., Skiendzielewski J. (1977). Myoma in association with Mayer-Rokitansky-Kuester syndrome. *American Journal of Obstetrics and Gynecology*.

[8] Rhee C. S., Kim J. S., Woo S. K., Suh S. J. (1999). MRI of round ligament leiomyoma associated with Mayer-Rokitansky-Kuster-Hauser syndrome. *Abdominal Imaging*.

[9] Tsin D. A., Waters T. K., Granato R. C. (2000). Laparoscopic myomectomy in a patient with Mayer-Rokitansky-Kuster-Hauser syndrome. *The Journal of American Association of Gynecologic Laparoscopists*.

[10] Edmonds D. K. (2003). Multiple fibroids in a postmenopausal woman with Mayer Rokitansky Kuster Hauser syndrome. *Journal of Pediatric and Adolescent Gynecology*.

[11] Jadoul P., Pirard C., Squifflet J., Smets M., Donnez J. (2004). Pelvic mass in a woman with Mayer-Rokitansky-Kuster-Hauser syndrome. *Fertility and Sterility*.

[12] Deligeoroglou E., Kontoravdis A., Makrakis E., Christopoulos P., Kountouris A., Creatsas G. (2004). Development of leiomyomas on the uterine remnants of two women with Mayer-Rokitansky-Küster-Hauser syndrome. *Fertility and Sterility*.

[13] Lamarca M., Navarro R., Ballesteros M. E., García-Aguirre S., Conte M. P., Duque J. A. (2009). Leiomyomas in both uterine remnants in a woman with the Mayer-Rokitansky-Küster-Hauser syndrome. *Fertility and Sterility*.

[14] Fukuda J., Kumazawa Y., Fujimoto T., Tanaka T. (2010). Mayer-Rokitansky-Kustner Hauser syndrome complicated by either uterine leiomyoma or ovarian tumor. *The Journal of Obstetrics and Gynaecology Research*.

[15] Rawat K. S., Buxi T., Yadav A., Ghuman S. S., Dhawan S. (2013). Large leiomyoma in a woman with Mayer-Rokitansky-Kuster-Hauser syndrome. *Journal of Radiology Case Reports*.

[16] Kundu K., Cohen A. W., Goldberg J. (2014). Acute torsion of uterine remnant leiomyoma with Mayer-Rokitansky-Küster-Hauser syndrome. *Fertility and Sterility*.

[17] Vidyashree P. G., Muralidhar P. V., Jayaram N., Latha K. (2015). Mayer-Rokitansky-Kuster-Hauser syndrome with multiple leiomyomas. *International Journal of Gynaecolology and Obstetrics*.

[18] Kulkarni M. M., Deshmukh S. D., Hol K., Nene N. (2015). A rare case of Mayer-Rokitansky-Kuster-Hauser syndrome with multiple leiomyomas in hypoplastic uterus. *Journal of Human Reproductive Sciences*.

[19] Girma W., Woldeyes W. (2015). Leiomyoma arising from Mullerian remnant, mimicking ovarian tumor in a woman with MRKH syndrome and unilateral renal agenesis. *Ethiopian Journal of Health Science*.

[20] Sharma R., Guleria K., Suneja A., Bhaskaran S., Tanveer N. (2017). Giant leiomyoma with extensive myxoid degeneration in Mayer-Rokitansky-Küster-Hauser syndrome. *International Journal of Gynaecology and Obstetrics*.

[21] Amaratunga T., Kirkpatrick I., Yan Y., Karlicki F. (2017). Ectopic pelvic fibroid in a woman with uterine agenesis and Mayer-Rokitansky-Küster-Hauser syndrome. *Ultrasound Quarterly*.

[22] Blontzos N., Iavazzo C., Vorgias G., Kalinoglou N. (2019). Leiomyoma development in Mayer-Rokitansky-Küster-Hauser syndrome: a case report and a narrative review of the literature. *Obstetrics and Gynecology Science*.

[23] Jokimaa V., Virtanen J., Kujari H., Ala-Nissilä S., Rantanen V. (2020). A Mayer-Rokitansky-Kuster-Hauser patient with leiomyoma and dysplasia of neovagina: a case report. *BMC Women’s Health*.

[24] Albahlol I. A., Elshamy M., El-Hady H. A. F., Abd-Elwahab E. M. (2020). Leiomyomas in a case of Mayer-Rokitansky-Kuster-Hauser syndrome: case report. *European Journal of Obstetrics and Gynecology and Reproductive Biology*.

[25] Ibidapo-Obe O., Okudo J., Filani O. (2021). Incidental finding of leiomyoma in Mayer-Rokitansky-Kuster-Hauser syndrome. *Journal of Investigative Medecine High Impact Case Reports*.

[26] Qiu S., Xie Y., Zou Y., Wang F. (2021). Giant hysteromyoma after vaginoplasty in a woman with Mayer-Rokitansky-Küster-Hauser (MRKH) syndrome: case report and review of the literature. *The Journal of International Medical Research*.

[27] Jain N., Kriplani I., Sharma S., Hanumantaiya S., Kriplani A. (2022). Urinary retention unveiling deeply embedded multiple leiomyomas in women with Mayer-Rokitansky-Kuster-Hauser syndrome and its successful laparoscopic management: a case-report and literature review. *Journal of Surgical Case Reports*.

[28] Parra C. M., Shirazian T. (2022). Laparoscopic removal of bilateral uterine remnants for symptomatic unilateral leiomyomas in a patient with Müllerian agenesis. *Fertility and Sterility*.

[29] Mueller G. C., Hussain H. K., Smith Y. R. (2007). Müllerian duct anomalies: comparison of MRI diagnosis and clinical diagnosis. *American Journal of Roentgenology*.

[30] Hall-Craggs M. A., Williams C. E., Pattison S. H., Kirkham A. P., Creighton S. M. (2013). Mayer-Rokitansky-Kuster-Hauser syndrome: diagnosis with MR imaging. *Radiology*.

[31] Alsaggaf R., St George D. M. M., Zhan M., Pfeiffer R. M., Wang Y., Anderson L. A. (2019). Benign tumors in myotonic dystrophy type I target disease-related cancer sites. *Annals of Clinical and Translational Neurology*.

[32] Jinnai K., Sugio T., Mitani M., Hashimoto K., Takahashi K. (1999). Elongation of (CTG)n repeats in myotonic dystrophy protein kinase gene in tumors associated with myotonic dystrophy patients. *Muscle and Nerve*.

[33] Khajavi M., Tari A. M., Patel N. B. (2001). “Mitotic drive” of expanded CTG repeats in myotonic dystrophy type 1 (DM1). *Human Molecular Genetics*.

[34] Mueller C. M., Hilbert J. E., Martens W., Thornton C. A., Moxley R. T., Greene M. H. (2009). Hypothesis: neoplasms in myotonic dystrophy. *Cancer Causes and Control*.

[35] Zaitseva M., Holdsworth-Carson S. J., Waldrip L. (2013). Aberrant expression and regulation of NR2F2 and CTNNB1 in uterine fibroids. *Reproduction*.

